# Screening and analysis of genes expressed upon infection of broad bean with *Clover yellow vein virus *causing lethal necrosis

**DOI:** 10.1186/1743-422X-8-355

**Published:** 2011-07-18

**Authors:** Kenji S Nakahara, Hiroaki Kitazawa, Go Atsumi, Sun Hee Choi, Yuji Suzuki, Ichiro Uyeda

**Affiliations:** 1 Pathogen-Plant Interactions Group, Plant Breeding Science, Research Faculty of Agriculture, Hokkaido University, Sapporo 060-8589, Japan

## Abstract

*Clover yellow vein virus *(ClYVV) causes lethal systemic necrosis in legumes, including broad bean (*Vicia faba*) and pea (*Pisum sativum*). To identify host genes involved in necrotic symptom expression after ClYVV infection, we screened cDNA fragments in which expression was changed in advance of necrotic symptom expression in broad bean (*V. faba *cv. Wase) using the differential display technique and secondarily with Northern blot analysis. Expression changes were confirmed in 20 genes, and the six that exhibited the most change were analyzed further. These six genes included a gene that encodes a putative nitrate-induced NOI protein (*VfNOI*), and another was homologous to an *Arabidopsis *gene that encodes a glycine- and proline-rich protein GPRP (*VfGPRP*). We recently reported that necrotic symptom development in ClYVV-infected pea is associated with expression of salicylic acid (SA)-dependent pathogenesis-related (PR) proteins and requires SA-dependent host responses. Interestingly, VfNOI and VfGPRP expression was correlated with that of the putative SA-dependent PR proteins in ClYVV-infected broad bean. However, broad bean infected with a recombinant ClYVV expressing the VfGPRP protein showed weaker symptoms and less viral multiplication than that infected with ClYVV expressing the GFP protein. These results imply that VfGPRP plays a role in defense against ClYVV rather than in necrotic symptom expression.

## Findings

Virus infections induce a number of disease symptoms such as developmental abnormalities, chlorosis, and necrosis in plants. These symptom expressions not only result from disruption of cell activity by virus infection but also from the complex interplay between plant and virus. Although it is broadly true that symptom severity, e.g., latent to lethal, depends on both viral pathogenicity and plant susceptibility, elucidating how these symptoms are expressed is an enormous challenge to plant virologists.

Recent studies regarding expression analysis of a large number of genes with microarrays have shown that sets of defense-related genes are expressed upon infection of a susceptible plant with several different viruses [1,2], suggesting that even susceptible plants recognize virus infection and mount defense responses. Ironically, in several plant-virus pathosystems, close relationships between defense responses to virus infection and severe necrotic symptoms have been reported [3-7]; these defense responses are thought to involve a hypersensitive response (HR) and programmed cell death (PCD), which are mediated by recognition of virus infection through a resistance (R) gene, which encodes a nucleotide-binding-leucine-rich repeat (NB-LRR) protein that detects a specific viral avirulence (Avr) gene. Although the way in which these defense responses are associated with necrotic symptoms largely remains to be examined, one likely explanation is that when a plant bearing an R gene is infected with a virus possessing a corresponding Avr gene and fails to control the spread of the virus throughout the entire plant, HR-PCD would necessarily occur wherever the virus infects, resulting in systemic necrosis [4,8]. We have recently shown that the lethal systemic necrosis observed in legumes infected with *Clover yellow vein virus *(ClYVV) is one such case involving defense responses [9,10]. Therefore, it is hardly surprising that the screening of host genes whose expression is elicited by ClYVV infection identifies genes involved in defense responses related to HR-PCD. The objective of the screening is, however, aimed at understanding the molecular mechanism of necrotic symptom expression in legumes infected with ClYVV.

**Figure 1:**
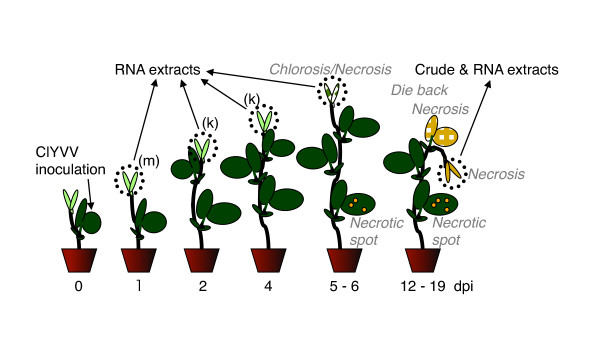
**Schematic representation of symptom expression in broad bean infected with ClYVV and inoculation and sampling schedule**. A first foliage leaf was mechanically or biolistically inoculated. Tip leaves were harvested for differential display and Northern blot analyses (RNA extracts) and Western blot and RT-PCR analyses (crude and RNA extracts) on the following days post inoculation (dpi).

A ClYVV culture derived from a plasmid pClYVV-Pst/CP clone, which carries full-length cDNA of ClYVV-No. 30 strain [11], was used for the screening of host genes. ClYVV usually induced necrotic symptoms in non-inoculated upper leaves of broad bean (*Vicia faba *cv. Wase) 5-6 days post-inoculation (dpi) and killed the plant following systemic induction of necrotic symptoms in 2 weeks (Figure 1). Western blot analysis with anti-ClYVV CP antibody revealed that ClYVV reached upper leaves at 4 dpi (data not shown).

To identify host genes whose expression changes are not just the outcome of necrotic symptoms but are a cause of the symptoms, gene expression was analyzed in broad bean before the development of ClYVV necrotic symptoms. Total RNAs were extracted from non-inoculated upper leaves of ClYVV and mock-inoculated plants at 1, 2, and 4 dpi with Trizol reagent (Invitrogen, Carlsbad, CA, USA), basically in accordance with the manufacturer's instructions (Figure 1), and their cDNA populations were compared using differential display. Fluorescent cDNAs were generated from the extracted total RNA by reverse transcription (RT) coupled with polymerase chain reaction (PCR) using an Enzyme Set-FDD and Fluorescence Differential Display Kit (TaKaRa, Shiga, Japan) according to the manufacturer's instructions. PCR products amplified using 96 different binary combinations with four fluorescent downstream primers and 24 upstream primers were fractionated in electrophoresis on 6% Long Ranger acrylamide gel (BioWhittaker Molecular Applications, Rockland, ME, USA) containing 8 M Urea. The fluorescent amplified cDNA fragments were visualized using an FMBIO II image analyzer (TaKaRa, Ohtsu, Japan).

We cut off 53 of the cDNA fragments (m fragments) specifically detected in samples from ClYVV-infected broad bean at 1 dpi and 210 of k fragments from samples at 2 and 4 dpi, when compared with mock-inoculated samples (Figure 1). These fragments were then re-amplified and cloned. When the nucleotide sequences of the clones were determined, 245 independent sequences among the 585 clones were identified. We secondarily screened 67 genes of the assertively selected sequences by Northern blot analysis using DIG-cRNA probes according to the manufacturer's protocol (Roche Diagnostics GmbH, Mannheim, Germany). Expression changes in 20 genes of ClYVV-infected broad bean were confirmed by at least two Northern blot analyses (data not shown). Six of the 20 genes were further analyzed because of their relatively large expression changes (Table 1).

**Table 1:** The six cDNA fragments whose expressions changed upon infection of broad bean with ClYVV

Flagments (deduced amino acid sequence)^1^	Homologous proteins (amino acid sequence)	Organisms	Identity (%)^2^	Accession
k47 (199 aa)	Glutathione S-transferase GST 16 protein (221 aa)	*Glycine max*	66.8	AF243371
k58 (79 aa)	Putative NOI protein, nitrate-induced protein (79 aa)	*Arabidopsis thaliana *	70.9	AY065260
k158 (202 aa)	GPRP (177 aa)	*A. thaliana*	55.5	NM_123903
	AtGPRP2 (179 aa)	*A. thaliana*	59	NM_118040
m2 (112 aa)	Putative senescence-associated protein (279 aa)	*Pisum sativum*	84.7	AB049724
m4 (74 aa)	Putative thaumatin-like protein (224 aa)	*Medicago truncatula*	65.4	AM943532
m18 (34 aa)	Quinone reductase family protein (205 aa)	*A. thaliana *	68.4	NM_118861

1. The full length mRNAs of k58 and k158 [DDBJ: AB615378, AB615379] were obtained by 5' and 3' RACE and others seem to be partial.

2. The identity of each fragment with the homologous protein was caluculated by GENETYX software (GENETYX, Tokyo, Japan).

**Figure 2:**
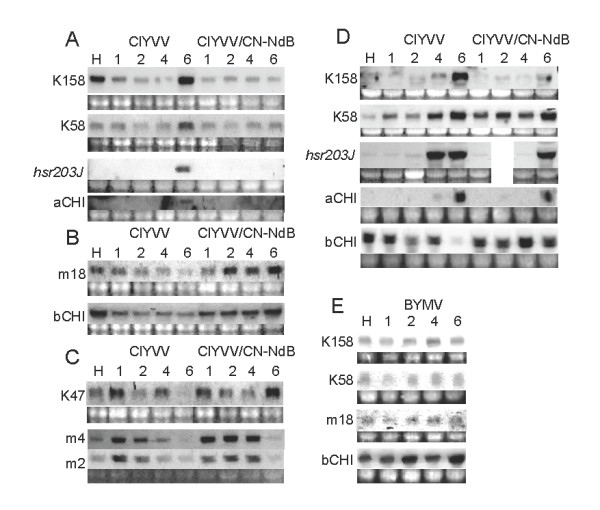
**Time course of expression of genes identified in this study and pathogenesis-related (PR) genes in ClYVV-infected plants**. mRNA accumulation was investigated by Northern blot analysis with DIG-labeled RNA probes. **(A, B, C) **RNAs were extracted from tip leaves of ClYVV- or ClYVV/CN-NdB-inoculated broad beans for Northern blot analysis (Figure 1). Hybridized probes were chemiluminescently detected with CDP-star reagent (New England Biolabs, Beverly, MA, USA). **(D) **The same experiment as in A, B, and C was performed with a pea line, PI 343958. **(E) **The same experiment as in D was performed with BYMV, which did not cause necrotic symptoms, but did result in mosaic symptoms in inoculated pea PI 343958.

Further analyses focused on the involvement of these genes in necrotic symptom expression. We recently found that necrotic symptom development in ClYVV-infected pea was associated with expression of genes encoding pathogenesis-related (PR) proteins and required salicylic acid (SA)-dependent host responses [9]. Consistent with this report [9], expression of *acidic chitinase *(*aCHI*) and *hsr203J *genes, which were putatively dependent on SA accumulation and cell death, respectively, was induced in leaves infected with ClYVV at 6 dpi (Figure 2A), and was therefore correlated with necrotic symptom expression in broad bean (Figure 1). In contrast, expression of the *basic chitinase *(*bCHI*) gene, which was putatively dependent on ethylene signaling, was negatively correlated with necrotic symptom expression (Figure 2B). Then, when expression of the six genes identified in this study was compared with that of the PR genes in ClYVV-infected broad bean, k58 and k158 showed similar expression patterns to *aCHI *and *hsr203J*, and were thus correlated with necrotic symptom expression (Figure 2A). Consistently, k58, k158, *aCHI*, and *hsr203J *were not elicited in broad beans inoculated with a ClYVV mutant derived from an infectious plasmid clone pClYVV/CN-NdB (ClYVV/CN-NdB) [12] that did not cause necrosis, but did cause weak chlorosis in broad bean. On the other hand, m18 expression was similar to that of *bCHI *and was negatively correlated with necrotic symptoms (Figure 2B). The other three genes, k47, m2, and m4, were elicited by ClYVV infection at 1 dpi, without reference to necrotic symptoms at earlier times. However, the expression of k47 was negatively correlated with necrotic symptoms at 6 dpi (Figure 2C).

The correlation of k58 and k158 expression with necrosis was further examined in a different host, pea (*Pisum sativum*). When pea line PI 343958 expressed necrotic spots under either ClYVV or ClYVV/CN-NdB infection [9,10], k58 and k158 expression, as well as expression of *aCHI *and *hsr203J*, was also elicited by either infection (Figure 2D). Nevertheless, k58 and k158 were not elicited in the same pea line (PI 343958) inoculated with *Bean yellow mosaic virus *(BYMV)-CS [13], which is another potyvirus closely related to ClYVV in terms of genome nucleotide sequences and host range, and does not cause necrosis, but does result in mosaic symptoms in pea (Figure 2E). Taken together, k58 and k158 expression was constantly correlated with *aCHI*, *hsr203J*, and necrotic symptom expression in legumes infected with ClYVV, raising the possibility of their involvement in the SA-dependent defense responses required for necrotic symptom expression.

**Figure 3:**
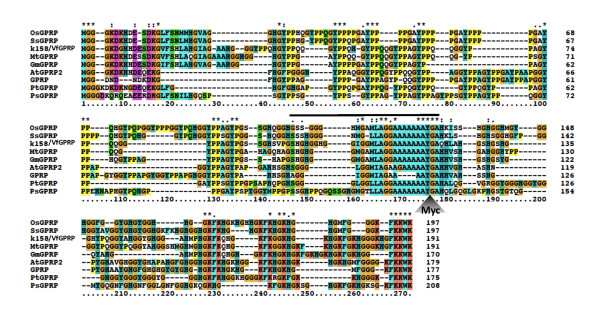
**Alignment of deduced amino acid sequences of GPRPs in diverse plants**. The GPRP sequences of *Oryza sativa *(OsGPRP) [DDBJ:NM_001061018], *Sporobolus stapfianus *(SsGPRP) [DDBJ:AJ242804] [16], broad bean (k158/VfGPRP, this study) [DDBJ:AB615379], *Medicago truncatula *(MtGPRP) [DFCI Medicago Gene Index TC:TC146460], *Glycine max *(GmGPRP) [DDBJ:BT089628], *Arabidopsis thaliana *(AtGPRP2), DDBJ:NM_118040], *A. thaliana *(GPRP) [DDBJ:NM_123903] [14], *Populus trichocarpa *(PtGPRP) [DDBJ:XM_002328813], and *Picea sitchensis *(PsGPRP) [DDBJ:EF083838] were aligned using the Clustal × program. The lined sequence was the central hydrophobic domain tentatively important for anchoring GPRP to a membrane [14]. A k158/VfGPRP mutant (VfGPmycRP) used in Figure 4 was made by inserting a Myc tag sequence, EQKLISEEDL, into the position indicated by a Myc-labeled arrowhead.

In this study, the involvement of k158 in necrotic symptom expression in broad bean was examined. An 860-bp full-length cDNA of k158 was obtained by conventional 5' and 3' RACE methods and its nucleotide sequence was determined [DDBJ: AB615379]. A BLAST search with the sequence found that the k158 gene product had 55.5% identity at the amino acid sequence level with a glycine- and proline-rich protein (GPRP) in *Arabidopsis thaliana *[14]. GPRP is well conserved in plants, including rice, soybean, poplar, and spruce (Figure 3). Conserved motifs among GPRPs were found throughout the amino acid sequence: MGG at the N-terminal, YPP repeats, a central hydrophobic domain tentatively required for anchoring GPRP to a membrane [14], HG and YG repeats, and FKKWK at the C-terminal. Taking the sequence identity and conserved motifs into account, we conclude that k158 is the broad bean homolog of *GPRP *(*VfGPRP*). The *VfGPRP *association with necrotic symptoms was examined by ectopic expression of VfGPRP protein in ClYVV-infected cells using a recombinant infectious plasmid pClYVV/VfGPRP derived from the virus expression vector pClYVV [11]. We also made another recombinant pClYVV/VfGPmycRP, which expressed a mutant VfGPRP protein with a Myc-tag peptide inserted into the central hydrophobic domain to potentially disrupt VfGPRP function (Figure 3).

**Figure 4:**
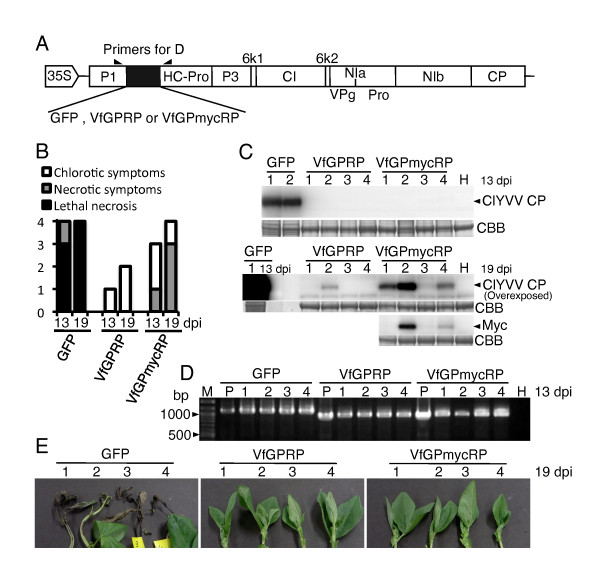
**Effect of ectopically expressed GPRP on ClYVV virulence in infected broad bean**. **(A) **Each of the recombinant ClYVV vectors possessing GFP, VfGPRP, or VfGPmycRP between P1 and HC-Pro cistrons was biolistically inoculated into broad bean leaves of four plants. **(B) **The reactions of broad beans inoculated with the recombinant ClYVV vectors, pClYVV/C3-S65T (GFP), pClYVV/VfGPRP (VfGPRP), or pClYVV/VfGPmycRP (VfGPmycRP), were evaluated at 13 and 19 days post-inoculation (dpi). The vertical axis indicates the number of plants showing the indexed symptoms. **(C) **The coat protein (CP) of ClYVV was detected in extract from a third upper leaf from the inoculated leaf by Western blotting with anti-CP polyclonal antibodies at 13 and 19 dpi. Since an extract from ClYVV/C3-S65T was no longer available at 19 dpi because of lethal necrosis, the extract from 13 dpi was applied for comparison of CP accumulation. VfGPmycRP protein was also detected using anti-Myc monoclonal antibody (Sigma-Aldrich, St Louis, MO, USA). Lane H is extract from a healthy broad bean leaf. CBB stained gels are shown as loading controls. **(D) **Viral genome cDNA including that of transgenes was detected in RNA extracts from the same leaves as used for Western blotting by reverse transcription-coupled PCR (RT-PCR) using primers 5'-GATGTACACGTGTGTCCAATGTCTTTTGG-3' and 5'-CTTATGGCATGCACATAATTGTTAACC-3', whose positions on the viral genome are shown in A. Lane P is PCR products amplified from the parental ClYVV vectors using the same primer pair. Lane H is the RT-PCR product with RNA extract from a healthy broad bean leaf. Lane M is a 100-bp ladder. **(E) **Photographs of tips of plants infected with recombinant ClYVVs.

These two plasmids and pClYVV/C3-S65T expressing GFP in infected cells [15] were biolistically inoculated into broad bean to compare their virulence (Figure 4A). ClYVV/C3-S65T caused lethal necrosis in infected broad beans about 2 weeks after inoculation and thus seems to have comparable virulence to wild-type ClYVV (Figure 4B and 4E). Compared with the ClYVV/C3-S65T-infected plants, drastically attenuated symptoms were observed in plants infected with ClYVV/VfGPRP. Additionally, accumulation of ClYVV/VfGPRP CP was far lower than that of ClYVV/C3-S65T (Figure 4C). RT-PCR nevertheless detected viral genomic cDNA fragments containing a transgene of an expected size in all tested RNA extracts from upper leaves (Figure 4D), indicating that ClYVV/VfGPRP at least infected and spread systemically and its progeny retained VfGPRP. Western blotting with anti-Myc antibody confirmed the expression of the VfGPmycRP protein in infected cells (Figure 4C). Taken together, these results suggest that VfGPRP represses ClYVV multiplication and/or spread in infected plants leading to attenuated symptoms. We cannot rule out the possibility of a *cis *effect of the inserted VfGPRP nucleotide and amino acid sequences on replication of the viral genome and/or translation of viral genes. However, the attenuated virulence was partly reversed by the additional insertion of a Myc tag peptide into the hydrophobic domain of VfGPRP on the recombinant ClYVV (ClYVV/VfGPmycRP; Figure 3, 4B, C, D and 4E), suggesting that the function of VfGPRP rather than its nucleotide and amino acid sequence is responsible for attenuation of ClYVV virulence. It is hard to obtain a mutant or transgenic broad bean in which VfGPRP expression is modified. Instead, we are preparing transgenic tobacco plants in which GPRP is overexpressed or knocked down by double-stranded RNA-mediated RNA silencing. We are now curious as to how GPRP is involved in not only the symptom expression of tobacco infected with poty- and other viruses but also internal viral replication and spread.

## Competing interests

The authors declare that they have no competing interests.

## Authors' contributions

IU designed and coordinated the study. HK and YS screened the broad bean genes using differential display techniques. HK performed cloning and sequencing of the identified genes and analyzed their expression. HK, GA, SHC, and KSN investigated the involvement of the identified genes in viral virulence in broad bean. KSN wrote the manuscript. All authors read and approved the final manuscript.
